# Ventilation failure after endotracheal intubation in an infant with abdominal compartment syndrome: A case report

**DOI:** 10.1002/ccr3.8424

**Published:** 2024-01-08

**Authors:** Yifan Fu, Zhen Luo

**Affiliations:** ^1^ Department of Anesthesiology, West China Hospital Sichuan University Chengdu China

**Keywords:** anesthesia, general, compartment syndromes, decompression, surgical, hypoventilation, infant, intra‐abdominal hypertension, pediatric emergency medicine

## Abstract

Intra‐abdominal hypertension and abdominal compartment syndrome (ACS) are distinct clinical stages of pathology caused by increased intra‐abdominal pressure, which may lead to respiratory and circulatory dysfunction in children and is associated with high pediatric mortality. An emergency exploratory laparotomy was planned for an infant with ACS. After induction of anesthesia and endotracheal intubation, the patient developed ventilation failure and any management was ineffective. Ventilation was resumed after a race against time abdominal decompression by the surgical team. Abdominal decompression is the primary treatment to relieve respiratory and circulatory failure in children with ACS.

## 1 INTRODUCTION

Intra‐abdominal hypertension (IAH) or abdominal compartment syndrome (ACS) occurs in almost 10% of children in neonatal and pediatric intensive care units.^1^ IAH and ACS are different clinical stages of pathological conditions caused by increased intra‐abdominal pressure (IAP) and are associated with high mortality in pediatrics.^2^ Normally, IAP is nearly equal to or slightly above ambient pressure. IAH is typically defined as abdominal pressure greater than or equal to 12 mmHg. ACS is a clinical syndrome characterized by a non‐physiological, progressive, and sharp increase in IAP caused by different factors, resulting in functional damage of intra‐abdominal organs and related extra‐abdominal organ systems. It is defined as sustained IAP exceeding 20 mmHg. In some cases, surgical decompression should be performed in a timely manner after the identification of ACS.^3^ However, anesthetic management of these patients is complicated by respiratory and circulatory dysfunction due to IAH. We report a case of an infant with ACS who failed positive pressure ventilation after endotracheal intubation.

An informed consent form was signed by the parents of the infant for the case reports. Ethics Committee approval is not required for individual case reports from West China Hospital, Sichuan University. This manuscript was prepared following the CARE guidelines (https://www.care‐statement.org).

## 2 CASE REPORT

A 2‐month‐old male (corrected gestational age: 40 weeks) weighing 2 kg was admitted to our hospital with abdominal distension for 2 months. He was born prematurely at 30^+2^ weeks and developed abdominal distension after birth. One day before admission his condition worsened with persistent vomiting, aggravated abdominal distension and cessation of defecation. Computed tomography (CT) of the abdomen showed distended intestines with multiple air‐fluid levels and bowel shadows in the left inguinal region. CT of the chest showed double pneumonia with partial consolidation. Admission diagnosis was (1) abdominal distension: Hirschsprung's disease?; (2) bilateral pneumonia; (3) sepsis; and (4) left inguinal hernia. An exploratory laparotomy was scheduled for the day after admission.

**FIGURE 1:**
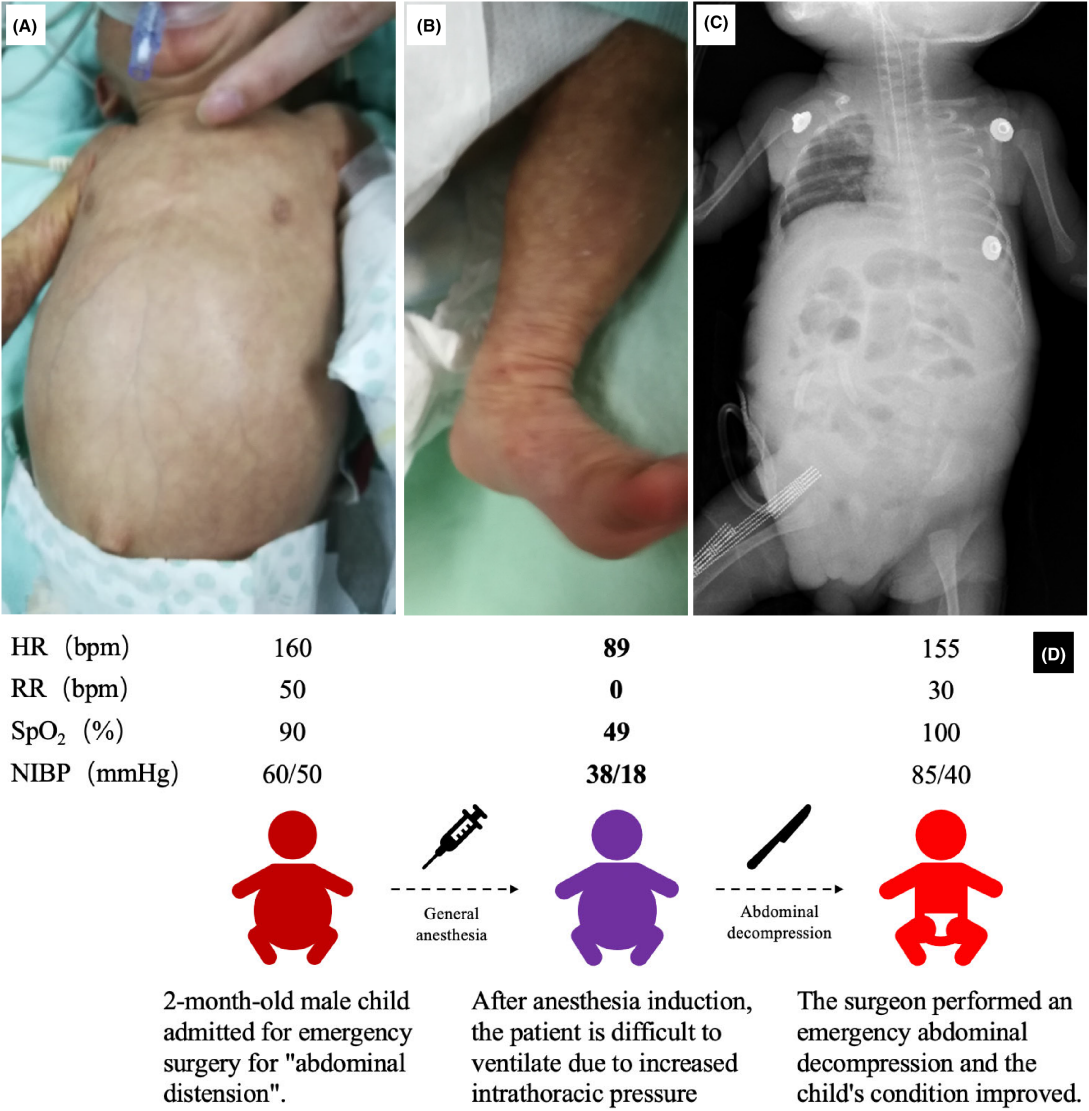
Patient clinical information. The child entered the operating room with a distended abdomen (A) and mottled limbs (B). The child's abdominal gas remained noticeable on the first postoperative day (C). The whole incident has been briefly described (D).

The following day, the infant presented to the operating room in an apathetic state with a large abdominal distension (Figure 1A), abdominal wall tightness and mottled lower extremities (Figure 1B). He had a tachycardia of 160 bpm, tachypnea of 50 bpm and his pulse oximetry was 90%, and his noninvasive blood pressure is 60/50 mmHg. He had already received a gastric tube and was undergoing gastrointestinal decompression.

Anesthesia was induced by sevoflurane inhalation. The concentration of sevoflurane was gradually increased from 1% to 2.5%, while midazolam 0.1 mg and fentanyl 2 μg were administrated intravenously. Spontaneous breathing was maintained throughout the induction. After an intravenous injection of 5 mg propofol, a 3.0 # cuffed endotracheal tube was inserted at a depth of 8 cm using a GlideScope. Ventilation became very difficult after intubation, with no chest movement. The ETCO_2_ waveform was not displayed, and the oxygen saturation dropped to 70%. First, insufficient depth of anesthesia was suspected, so propofol 5 mg and succinylcholine 5 mg were given immediately, but there was no improvement. Second, severe bronchospasm was suspected. Adrenaline 5 μg was injected intravenously. Ventilation was impossible and the SpO_2_ continued to drop to 49% and the heart rate dropped from 120 to 89 bpm, with 38/18 mmHg NIBP. The surgeons were then asked to open the abdomen immediately, as high abdominal pressure was thought to be the cause. Fortunately, ventilation improved immediately after abdominal decompression. An increase from 0 to 42 appeared on the ETCO_2_ waveform. Oxygen saturation returned to 100% and HR increased to pre‐crisis levels, and his NIBP up to 85/40 mmHg. The whole incident has been briefly described in Figure 1D. The surgical team performed colonic decompression and terminal ileostomy. The patient was diagnosed with Hirschsprung's disease after surgery. The postoperative course was uneventful. Unfortunately, the diagnostic basis is incomplete, and there is no etiologic examination for sepsis. After 7 days in the intensive care unit, the patient was transferred to the general ward.

## 3 DISCUSSION

Despite the extensive literature on the diagnosis and management of ACS in recent years, few studies have addressed the anesthetic management of pediatric patients.^4^ A major challenge in these patients is the high risk of anesthetic induction. First, elevated IAP increases intrathoracic pressure through the abdominal distension and elevated diaphragm, leading to decreased functional residual volume, compressive atelectasis and ventilation perfusion mismatch, resulting in impaired respiratory function and decreased oxygen reserve; Second, increased intrathoracic pressure decreases preload by compressing the inferior vena cava and increases afterload by compressing the aorta, leading to cardiac insufficiency; third, these patients are at high risk for reflux aspiration.

In this case, the presence of sepsis exacerbated the patient's respiratory and circulatory compromise, further complicating the clinical situation. Based on the above considerations, we decided to maintain spontaneous breathing of anesthesia induction and prepared for rescue. At the same time, we also considered the possibility of ventilation failure after induction and asked the surgeon to prepare for surgery in advance.

Unfortunately, the infant developed ventilation failure after endotracheal intubation. We first deepened the anesthesia by administering propofol and succinylcholine. Epinephrine was given later, but ventilation was still not possible and the infant was at imminent risk of cardiac arrest. At this point, the only hope of saving the patient was to perform emergency abdominal decompression.

Yang et al. once reported a case of cardiac arrest in a neonate with ACS who was successfully resuscitated by needle decompression.^5^ The diagnosis of that patient was gastric perforation with free air in the abdominal cavity. Needle decompression successfully relieved the distension and saved the infant. However, the increased abdominal pressure in our patient was due to intestinal swelling and intestinal gas accumulation, with no apparent free air in the abdominal cavity. Therefore, emergency needle decompression was not feasible in our patient. In this critical situation, the surgical team opened the abdomen in the shortest time and reduced the intra‐abdominal pressure, which immediately improved ventilation and prevented further hypoxia and heart failure. It also confirmed that the failure of ventilation was due to increased intrathoracic pressure transmitted by abdominal distension.

## 4 CONCLUSION

In conclusion, it is a challenge for anesthesiologists and surgeons to manage children with IAH or ACS who require surgical intervention. Abdominal compliance in patients with ACS is beyond the limits of self‐regulation, and the use of any anesthetic drug in a critically ill patient will upset the balance that the patient has worked so hard to maintain, leading to increased intrathoracic pressure and thus affecting the respiratory and circulatory system, especially with muscle relaxant drugs. For such patients, the anesthetist and surgeon need to determine the appropriate plan before surgery. Effective gastrointestinal decompression should be performed prior to anesthesia whenever possible, and if gastrointestinal decompression is ineffective or anesthetic induction is considered first, a surgeon capable of performing open gastrointestinal decompression alone is required on standby.

## AUTHOR CONTRIBUTIONS

**Yifan Fu:** Writing – original draft; writing – review and editing. **Zhen Luo:** Supervision; writing – original draft; writing – review and editing.

## FUNDING INFORMATION

Not applicable.

## CONFLICT OF INTEREST STATEMENT

The authors report no conflict of interest. The data used to support the findings are included within the case report.

## ETHICS STATEMENT

Ethics Committee approval is not required for individual case reports from West China Hospital, Sichuan University. An informed consent form was signed by the parents of the infant for the case reports.

## CONSENT

Written informed consent for publication of patient details of this case report was obtained from the patient's next of kin.

## Data Availability

The data used to support the findings are included within the case report.
